# Conduct and Implementation of Personalized Trials in Research and Practice

**DOI:** 10.1162/99608f92.901255e7

**Published:** 2022-09-08

**Authors:** Richard L. Kravitz, Naihua Duan

**Affiliations:** 1 Department of Internal Medicine, University of California, Davis; 2 Sacramento, California, United States of America; 3 Division of Mental Health Data Science, Department of Psychiatry, Irving Medical Center,Columbia University; 4 New York City, New York, United States of America

**Keywords:** personalized trial, randomized controlled trial (RCT), heterogeneity of treatment effects (HTE), individualized treatment effect (ITE), blinding, washout

## Abstract

The mainstay of evidence development in medicine is the parallel-group randomized controlled trial (RCT), which generates estimates of treatment efficacy or effectiveness for the average person in the trial. In contrast, personalized trials (sometimes referred to as ‘single-person trials’ or ‘N-of-1 trials’) assess the comparative effectiveness of two or more treatments in a single individual. These single-subject, randomized crossover trials have been used in a scattershot fashion in medicine for over 40 years but have not been widely adopted. An important barrier is the paucity of strong evidence that personalized trials improve outcomes. However, the principal impediment may have less to do with proof of efficacy than with practical aspects of design and implementation. These include decisions about treatment regimen flexibility, blinding, and washout periods as well as organizational, clinician, and patient-level challenges. After reviewing the essential elements of personalized trials, this article addresses these speed bumps and fundamentally asks, ‘Why have personalized trials not been more widely adopted, and how can they be made more readily deployable and useful?’ The article concludes by suggesting ways in which emerging technologies and approaches promise to overcome existing barriers and open promising vistas for the next generation of personalized-trial researchers and practitioners.

## 1. Introduction

Parallel-group randomized controlled trials (RCTs) have made an enormous contribution to health and health care. They randomly assign patients to two or more treatment arms; the comparisons are between groups. When properly designed and conducted, these trials provide unbiased estimates of the ‘average treatment effect’ for participants in the trial. Evidence generated via this approach is surely better than the ‘eminence-based medicine’ of prior eras. However, the typical patient in a trial is often surprisingly different from average, especially with respect to prognosis (Kent et al., 2010). For this reason, clinical researchers and statisticians have avidly sought methods for estimating the effects of different treatments on individuals—that is, to account for heterogeneity of treatment effects (Kravitz et al., 2004). One approach, ideal in some circumstances and entirely unworkable in others, is the personalized (N-of-1) trial.

Personalized trials are randomized crossover trials conducted in a single patient. Such trials are a subset of single-case designs, which “study intensively the process of change by taking many measures on the same individual subject over a period of time” (n.d.-a). Single-case designs have played an important role inside and outside of medicine for many generations (R. D. Mirza et al., 2017). However, randomized crossover trials in an individual (i.e., personalized trials) have a shorter history (Guyatt et al., 1986)(n.d.-b)(n.d.-c).

Personalized trials chiefly aim to guide treatment for the individual. Their singular advantage is the ability to directly estimate the individual treatment effect (ITE): the difference (or ratio) of outcomes between one treatment and another in a given person. The ‘treatment’ and its comparator can be a drug, a dietary supplement, a short-acting procedure, a behavior, a placebo, or no treatment at all. By switching treatments in a defined sequence over time, an individual can compare outcomes while on alternative regimens, thus providing a direct estimate of how well a given treatment works *for her*.

To date, adoption of personalized trials has been modest. (Gabler et al., 2011) found 108 personalized trials (or trial series) published between 1985 and 2011, while (Punja et al., 2016) independently found 100 reports appearing between 1950 and 2013. In contrast, there have been nearly 400,000 RCTs (mostly of the parallel group type) reported in PubMed since 2000.

The relatively slow rate of uptake has tended to disappoint personalized trial proponents (Vohra, 2016). One explanation is that such trials have simply not delivered on their promise of improving clinical outcomes; they represent “another instance of a beautiful idea being vanquished by cruel and ugly evidence” (Reza D. Mirza & Guyatt, 2018). However, others argue that the concept has not been sufficiently tested (Kravitz et al., 2019). In addition, qualitative research with patients and clinicians suggests that many have never heard of the approach, have little sense of how to implement such trials in the context of a busy practice, or are skeptical as to whether the putative benefits (e.g., enhanced patient engagement in care, potentially improved clinical decision-making) are worth the costs and burdens (Cheung et al., 2020)(Kravitz et al., 2020)(Kronish et al., 2017)(Moise et al., 2018). A quote by a physician-participant in an interview study is particularly apt: “Well, I personally would be interested in that, but I think one of the biggest limitations . . . is time and time constraints” (Kravitz et al., 2009).

Personalized trials may yet find their place in the clinical and wellness landscape for two reasons. First, new developments in biostatistics, health informatics, and information technology are helping to streamline and automate many personalized trial functions. These innovations allow people to design their own trials and more readily collect, organize, analyze, and interpret personalized trial data. In particular, mobile devices combined with a robust backend may partially obviate the need for personalized trial ‘services’ established at the organizational, state, or national level (Chalmers et al., 2019).

**Figure 1.**
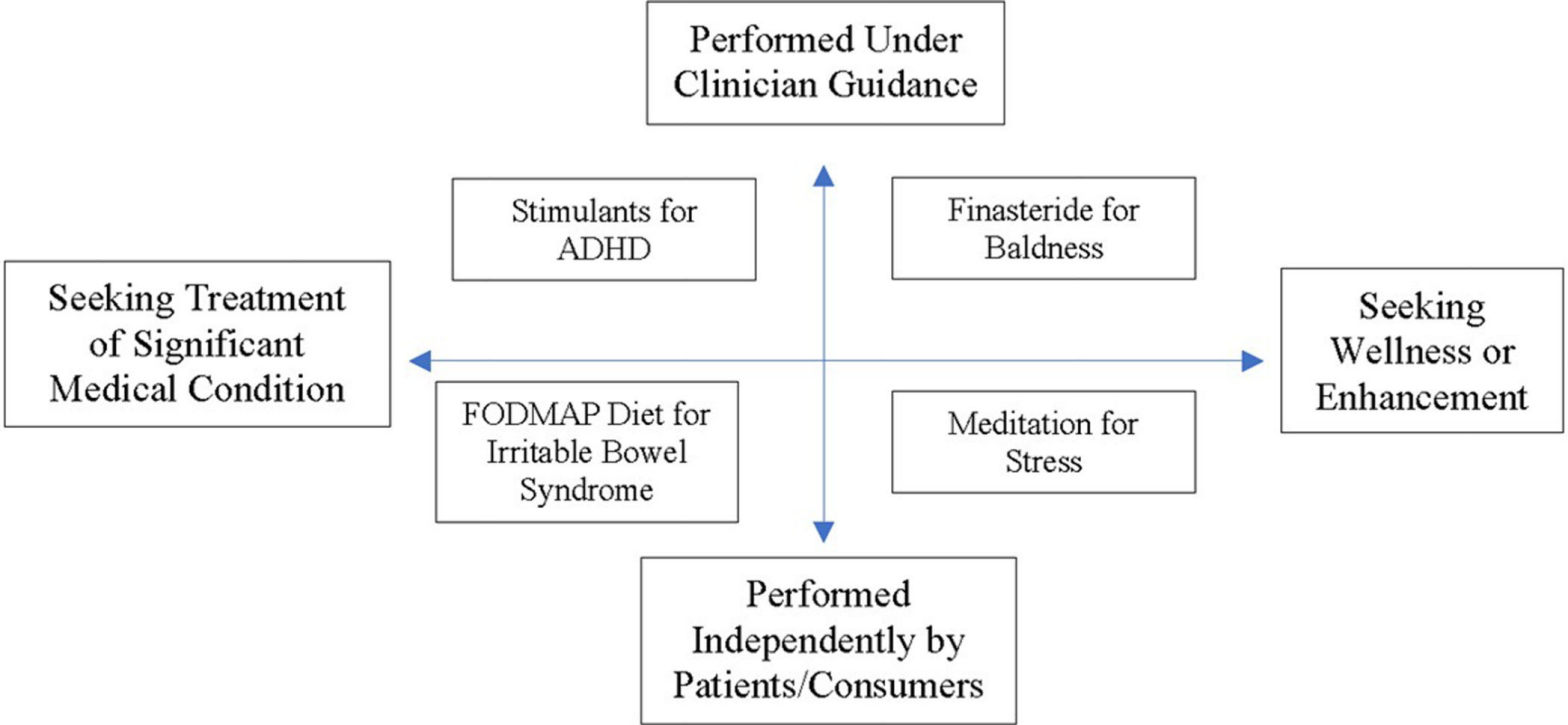
The landscape of personalized trials. The landscape of personalized (N-of-1) trials is represented along two dimensions. The horizontal axis depicts the spectrum of health needs, ranging from significant medical conditions (on the left) to health promotion and disease prevention (on the right). The vertical axis represents the degree of professional (clinical) supervision required (minimal at bottom, intensive at top). ADHD = Attention Deficit-Hyperactivity Disorder; FODMAP = fermentable oligosaccharides, disaccharides, monosaccharides and polyols

Second, inspired by investigators in the behavioral sciences and by the quantified self movement (Swan, 2013), an increasing number of personalized trials are being conducted among people seeking to mitigate symptoms or enhance wellness with or without the guidance of a licensed health professional. (In this context, personalized trials represent a rigorous extension of self-tracking, itself a growing trend with implications for both self-care and health enhancement. (Jin et al., 2022)). Along these lines, the personalized trial landscape can be conceptualized as a Cartesian plane with the two axes representing the underlying purpose (treatment versus health enhancement) and the need for clinical supervision (performed with professional guidance versus independently; Figure 1).

This article covers the essential elements of personalized trials, explores barriers to uptake and use, and discusses emerging technologies and approaches that may facilitate expanded use.

## 2. Essential Elements of Personalized Trials

Regardless of where they fall within the landscape depicted in Figure 1, personalized trials have universal requirements. Some of these requirements are *technical* and guide the selection of subjects, health conditions, and interventions; the means by which trials are conducted; and the way data are analyzed and aggregated. Others are *social* and *organizational*; they bear on how participants are recruited, enrolled, and supported. Even when trials are conducted by individual patients/consumers acting independently of the health care system (bottom half of Figure 1), there is still a need for both technical support (e.g., in the form of ‘apps’) and social support (often taking the form of online discussion groups).

### 2.1. Technical Requirements

The technical requirements for personalized trials include criteria related to the population, health condition, treatment, and design and analysis of the trials themselves (Table 1). Two additional elements (i.e., blinding and washout) may also be necessary depending on the specific circumstances.

#### 2.1.1. Technical Requirements Related to the Population, Health Condition, and Treatment

1. *Substantial heterogeneity of treatment effects (HTE)*. In qualitative terms, if HTE is small, then most patients respond to treatment in the same way, so one may then assume that average effects derived from parallel-group trials accurately signal what a given individual might expect. This would make personalization unnecessary. More quantitatively, HTE can be defined as the standard deviation of the individual treatment effects, which is proportional to the pooled standard deviation of the outcome, *SD*, and the correlation between the outcome of individuals receiving each of two treatments, ρ. This is given by the formula $\sqrt{2}SD\;\sqrt{1-c\rho }$, where *c* is a correction factor representing the ratio of the geometric mean of the outcome variances across arms to the arithmetic mean of the same variances. Since ρ usually ranges between 0 and 1 and the correction factor is typically near 1, HTE can range from close to 0 (i.e., when the outcomes on the treatment and comparator are perfectly correlated) to $\sqrt{2}$ *SD* (i.e., when the outcomes on the two treatments are completely uncorrelated; (Kravitz et al., 2004)(n.d.-d).
2. *A health condition that is chronic, relatively stable, and monitorable with a validated patient-reported outcome measure (PROM) or biomarker.* Acute conditions will tend to resolve (or progress) before personalized trials can be completed. Rapidly progressive or fatal conditions are likewise unsuitable. Acute severe coronavirus disease 2019 (COVID-19), for example, is a poor platform for such trials because the disease may kill the patient before multiple treatment crossovers can be accomplished. In contrast, chronic symptoms following COVID-19 infection (‘long COVID’) is an ideal target for personalized trials, as the typically prolonged course permits many treatment switches. Finally, the statistical reliability of single-patient, multiple-crossover trials increases the more often outcomes are assessed. This is why many personalized trials take measures daily or more often. The most common method for obtaining serial outcome measurements is through direct patient or proxy reports (i.e., surveys). Sometimes, a clinical measurement (e.g., blood pressure) or laboratory value (e.g., blood glucose) can be used as a proxy outcome. Increasingly, personalized trials have begun to incorporate outcomes data obtained during daily life via mobile devices (e.g., daily steps, sleep, social interactions).
3. *Treatments that have rapid onset and modest carry-over effects.* Because, in the sense we use the term, personalized trials require at least two treatment switches (e.g., BAAB or ABBA) and multiple outcome measurements, such studies can stretch on for some time and potentially try the patience of participants. The ideal study treatment will take effect quickly and dissipate rapidly. An excellent clinical example might be use of inhaled levodopa (Inbrija^®^) versus oral, immediate-release levodopa-carbidopa (Sinemet^®^) for ‘off-periods’ in Parkinson’s disease. Both of these agents take effect within minutes and wear off after a few hours. In contrast, given their extended biological half-life, bisphosphonates for osteoporosis would be a terrible candidate for personalized trials. Some personalized trial investigators have dealt with the problem of prolonged treatment effects by incorporating washout periods (i.e., sufficient time in between treatment switches for the initial treatment to wear off) or various analytic techniques that adjust for carryover (e.g., by downweighting outcome measurements obtained soon after a switch). We discuss the use of washouts in Section 2.1.3.

#### 2.1.2. Technical Requirements Related to Trial Design and Implementation

1. *Randomized or balanced treatment assignment.* In most conventional clinical trials, the unit of analysis is the individual participant. In personalized trials, the unit of analysis is a segment of time (i.e., hour, day, week, etc.). Put more clearly, in RCTs, people are randomized to treatments, but in personalized trials, treatments are randomized within people. Treatments must be allocated in a manner that minimizes bias, maximizes statistical information, and conveys credibility to participants and clinicians. This is usually achieved with an appropriate restricted randomization scheme. Unrestricted random assignment might result in sequences with poor credibility, validity, and efficiency such as AAAABBAA, which frontloads Treatment A and allocates 75% of the entire study period to this treatment. Therefore, many experts restrict random assignment so as to limit randomization to a subset of possible sequences with desirable statistical properties while conveying reasonable credibility to the end users. For example, a trial comparing two treatments with weekly switches lasting a total of four weeks could restrict the randomization to the following four allowable sequences: ABAB, ABBA, BAAB, and BABA. These sequences allocate half of the treatment segments to each treatment within each block of two consecutive time segments. The randomization can be restricted further to the following two allowable sequences: ABBA and BAAB. These sequences are more robust against the possibility of confounding with time trend than the sequences ABAB and BABA.
2. *Systematic assessment and collection of outcomes.* In personalized trials, systematic assessment of outcomes may well be the single most important design element. Two issues need consideration: (1) what data to collect and (2) how to collect them (n.d.-e). For most chronic conditions, many outcomes are potentially relevant; they may be condition-specific (e.g., pain intensity in chronic low back pain, diarrhea frequency in inflammatory bowel disease) or generic (e.g., health-related quality of life). The ideal measure is reliable, valid, and—especially for trials where the primary aim is to inform clinical care of the current patient rather than produce generalizable evidence or influence regulatory decisions—closely matched to the patient’s priorities (when the focus is on generalizable evidence or regulatory approval, use of reproducible measures is essential). When such measures are unavailable off the shelf, patients and clinicians must design their own or enlist a hybrid approach, such as the Measure Yourself Medical Outcome Profile (MYMOP; (Ishaque et al., 2019). Personalized trials can make use of the entire spectrum of data-collection modalities from surveys, diaries, medical records, and administrative data to newer technologies involving mobile devices and remote monitors.
3. *A framework for statistical analysis and feedback for decision making*. Once data are collected, the results need to be analyzed and presented to the relevant decision makers in an actionable form. Developers and users of personalized trials have three issues to consider: (1) should outcomes be combined, and how? (2) how should the data be presented? and (3) to what extent should various forms of prior knowledge be integrated into decision making? Separate measures retain clinical granularity, while composite measures distill complex information into fewer numbers or even a single number. Simple graphs are appealing to many patients but tend to ignore or downplay uncertainty. More complex graphs and tables might have allure for more sophisticated users, but could be hard for others to decipher and interpret. Some evidence suggests that combining simple graphs *and* verbal summary statements may have the widest reach (Whitney et al., 2018). Whether to customize the presentation, and how, is an important task for personalized trials, as well for the broader famework of personalized data science (see companion article in this issue, (n.d.-f)). Finally, within a Bayesian framework, results of personalized trials are more robust when bolstered by external evidence, whether from other similar personalized trials or the clinical literature.

#### 2.1.3. Optional Elements That May Be Required in Selected Circumstances

1. *Blinding.* This term generally refers to “keeping study participants, those involved with their management, and those collecting and analyzing clinical data unaware of the assigned treatment, so that they should not be influenced by that knowledge” (Day & Altman, 2000). Blinding of participants and clinicians in personalized trials can be challenging and is often unnecessary. Blinding is essential when there is a need to separate the biological activity of the treatment from nonspecific (placebo) effects. This is certainly the case in most parallel group drug and device trials as well as personalized trials conducted in series for the purpose of obtaining regulatory approval of a new therapeutic agent. However, in many personalized trials, participants are most interested in the *overall* effects of treatment, defined as the sum of specific and nonspecific effects. Therefore, blinding may be less important (and even counterproductive) in this context.
2. *Washout*. As noted above, a condition-treatment pair is ideally suited for the multiple-crossover approach of personalized trials when the condition is relatively stable (i.e., neither wildly fluctuating nor unrelentingly improving or deteriorating) and the treatment has a rapid onset and offset. However, many such condition-treatment pairs are suboptimal. When researchers are concerned that the effects of the treatment administered first may bleed over into the next observation period, their solution is often to introduce a washout period. Washouts may be ‘physical’ or ‘analytical’ (Hogben & Sim, 1953). In a physical washout, a period of time is permitted to elapse between treatments, and the interval depends on expected treatment duration. For pharmaceutical interventions, the washout interval would be an appropriate multiple of the elimination half-life. In addition to prolonging trial length, physical washouts introduce ethical problems, as patients are necessarily denied access to potentially effective treatment for the duration of the washout. In an analytical washout, treatments are administered sequentially without a break, but measurements are adjusted up or down (‘reweighted’) to account for what is known about the carryover and start-up effects, thus producing the equivalent of physical washout without unduly withholding treatments from patients. Analytic washouts cannot compensate for observation periods that are too short relative to the duration of action of the treatment.

### 2.2. Social and Organizational Requirements

In addition to these technical requirements, clinicians and clinical investigators interested in launching personalized trials need social and organizational support. Within health care settings, clinicians hoping to make personalized trials available to their patients must begin with a keen understanding of the indications, strengths, and limitations of the method; these trials are not for everyone. They should also be adequately committed to the process so as to not only convey their enthusiasm to patients but also weather the inevitable setbacks, delays, and ambiguities. Beyond their own personal commitment, clinician-investigators need support from organizational leaders and colleagues. While personalized trials may lower costs in the long run (Kravitz et al., 2008)(Pereira et al., 2005)(Scuffham et al., 2010), they can impose significant time demands and require ongoing investment in personnel and infrastructure. For example, (Scuffham et al., 2008) estimated the fixed cost of personalized drug trials at AU$23,280 for each protocol; this included staff costs for protocol development, funding applications, ethics agreements, preparation of forms and questionnaires, database development, and design and preparation of medication packs. (A single protocol could serve as the framework for personalized trials conducted in multiple individuals.) The variable (i.e., per-patient) costs were estimated at roughly AU$600, which included recruitment, administration, data collection and analysis, feedback, and 12-month follow-up of outcomes. Given the relatively low marginal costs of enrolling each additional patient, successful personalized trial programs create economies of scale. Organizational leadership must step up to not only provide the initial investment but also support clinician champions in bringing along colleagues who recruit additional patients.

Outside of health care settings, personalized trials need participants, and participants need a platform that makes participation easy. Investigators, meanwhile, must identify a target population, develop a marketing strategy, and encourage enrollment through social networks. As an example, in a recently published personalized trial series marketed to the general public, a multidisciplinary team used social media and an interview on the *Brian Lehrer Show* (on NPR.org) to recruit participants interested in trying out one of several simple behavioral interventions for promoting psychological well-being (Kravitz et al., 2020). They created a website with training videos, provided participants with a mobile app for reporting daily outcomes during intervention and control periods, and returned results via a personal web link.

Whether studies are conducted within or outside of health care settings, research suggests that many patients are natural enthusiasts for self-tracking but do not necessarily appreciate the benefits of randomized (or balanced) switching between treatments, and they are not always prepared to interpret even simple numerical or graphical results (Whitney et al., 2018). Therefore, in designing personalized trials, investigators need to account for patient preferences (Moise et al., 2018) and information-processing styles (Gigerenzer et al., 2007).

## 3. Barriers to Uptake and Use

Although personalized trials have many adherents and a few evangelists, implementation has been slow. As suggested earlier, a major reason is conflicting evidence; few RCTs have directly compared personalized trials to standard care, and most of those have produced marginally positive, equivocal, or unconvincing results. For example, in a study by (Mahon et al., 1996), personalized trials succeeded in convincing a large proportion of asthma patients taking theophylline without benefit to discontinue the medication. In a study of arthritis patients by (n.d.-g), N-of-1 patients had slightly better outcomes at substantially higher cost. Further, in an RCT by (Kravitz et al., 2018), chronic pain patients assigned to the N-of-1 group had slightly better pain interference and significantly enhanced medication-related shared decision-making. However, the primary outcome of pain interference was not statistically significant.

On the other hand, a substantial number of published case series support the feasibility and value of personalized trials for individual patients, and some argue that RCTs are an inappropriate testing ground for such trials because of the very nature of personalization (Vohra, 2016). As it stands, much of the ‘evidence’ supporting personalized trials derives from case series in which trial participation has been associated with (1) choosing a more personally effective treatment for long-term use, (2) choosing a safer or less-costly treatment for long-term use, or (3) continuing with an evidence-based treatment that *allegedly* causes side effects (Joy et al., 2014)(Nikles et al., 2006)(Nikles et al., 2007)(Yelland et al., 2007). The next generation of personalized trial researchers will further unravel the inherent heterogeneity of treatment effects (where personalized trials are the treatment) and identify which patients benefit and which do not.

Setting aside the question of ‘effectiveness’ of personalized trials for the average patient, what are the remaining barriers to their uptake? These may be broadly categorized as intrinsic or extrinsic. *Intrinsic factors* include elements of trial design that may or may not be essential but increase burden to investigator, clinician, or participant. *Extrinsic factors* include perceptions of benefit and cost as assessed by organizations, clinicians, and patients/participants.

### 3.1. Intrinsic Factors

The principal intrinsic (design) factor to consider is treatment regimen rigidity versus flexibility. In parallel group RCTs, treatment protocols are well-defined with limited opportunities for adjustment. For example, in cancer trials, a fixed-dose (i.e., mg/kg), two-drug chemotherapy regimen may be compared to a three-drug regimen with allowances for a 50% dose reduction in the event of certain side effects. Similarly, investigators interested in aggregating the results of N-of-1 series through meta-analysis will naturally prefer relatively rigid treatment regimens to simplify inferences about overall (average) treatment effects, HTE, and predictors of individual treatment effects. Although some regimen-related variation is manageable using network meta-analysis, the quest for generalizability will tend to favor uniform treatment regimens.

In contrast, the primary goal of personalized trials is to guide treatment for the individual. Since people differ in terms of their physiology, psychology, social determinants, and preferences, treatment regimens for comparison often need tailoring. For example, in the PREEMPT (Personalized Research for Monitoring Pain Treatment) Study, patients with chronic musculoskeletal pain were encouraged to make comparisons among any combination of eight treatment categories including acetaminophen, nonsteroidal anti-inflammatory drugs, short-acting opioids, and various non-pharmacological complementary and alternative treatments.

Other design factors (introduced earlier in this article as ‘optional elements’) are blinding and washout. Blinding may be essential when it is critical to exclude non–drug-related (nonspecific) benefits (i.e., placebo effect) or to investigate adverse effects (i.e., nocebo effect; (Herrett et al., 2021). At least one rating scale has incorporated blinding as a criterion for personalized trial quality (Tate et al., 2013). However, blinding may be impossible in some settings (e.g., with most behavioral interventions) and unnecessary in others (e.g., when the patient or clinician is interested in the sum of specific and nonspecific effects). Indeed, absent the need to generalize to other people or populations, blinding may be undesirable because it would preclude accounting for the sum of specific and nonspecific treatment effects in an individual. In addition, blinding increases costs and decreases regimen flexibility.

Washout periods—introduced to guard against carryover effects—have their own limitations (Duan et al., 2013). Patients and clinicians may be dissatisfied with the withholding of active treatments during the washout. If the treatment has a slow onset, a washout period can increase the delay before clinical effects are realized and thereby stretch out the duration of the trial.

### 3.2. Extrinsic Factors

Personalized trials targeted to clinical populations (see Figure 1) require the support of organizations (e.g., governments, health systems, hospitals, clinics, or practices). With robust support from organizational leadership, investigators can recruit clinicians and patients; hire pharmacists, statisticians, and database managers; overcome inevitable administrative hurdles; and amass the necessary resources to implement trials efficiently and effectively. If the organization is resistant, personalized trial implementation is much more difficult.

A major barrier to the incorporation of personalized trials into routine practice is the ongoing debate over whether these trials represent ‘research’ or merely a more rigorous approach to routine clinical care. If personalized trials are research, then the usual requirements (i.e., institutional review board [IRB] approval, written informed consent, and third-party monitoring) all apply. If they are simply an upgrade to clinical care, then authority devolves to the clinician and patient through a process of shared decision-making and oversight through applicable licensing and credentialling bodies. As (n.d.-h)have argued:

If the primary interest is to produce local knowledge to inform treatment decisions for individual patients, n-of-1 trials so conducted should be interpreted as *clinical* care, and in our view are not subject to the HHS protection of human subjects regulations. Alternatively, if the primary interest is to produce generalizable knowledge to inform treatment decisions for future patients, such n-of-1 trials should be interpreted as *human subjects research* and required to comply with the standards of such research.

Two sources of difficulty are worth noting. First, organizational leaders and ethics boards may not accept the premise that personalized trials are not research. Second, distinguishing between intent to produce local (i.e., individual patient) knowledge and generalizable (i.e., more broadly applicable) knowledge can be challenging. A widely held but erroneous belief is that intent to publish constitutes research (n.d.-i). However, disputed areas also present various issues. For example, what if the data from a patient undergoing a personalized trial to inform their own care will be aggregated with data from other patients undergoing similar trials for the purpose of assessing both average treatment effects and HTE? What if the individual’s data are used to inform the treatment of the next patient? What if some or all of the treatment options within a personalized trial are in common use for that indication but are not FDA-approved (i.e., they are used ‘off-label’)? (n.d.-j) provide a reasonable framework for sorting out some of these difficulties, but they have yet to be resolved.

These ethical and regulatory conundrums aside, organizational leaders simply may not see the value proposition in personalized trials. Given fixed and variable costs totaling US$1,000 or more per case, (n.d.-k) conclude that personalized trials will gain economic traction only when applied to clinical areas where treatment costs are high, serious side effects are prevalent, and infrastructure is adequate to support trials that are straightforward, efficient, and timely.

At the clinician level, the major barriers are both practical and relational (Kravitz et al., 2009). Practically speaking, many clinicians will conclude that personalized trials, at least for most patients, are simply not worth the time and effort. This conclusion is based partly on the judgment that therapeutic trials as used in customary practice are often ‘good enough’ and partly on the impression that many patients already struggle with adherence and self-monitoring and will ‘not do well’ with the extra demands entailed by multiple crossover trials. Of course, some of these concerns might be obviated by a more flexible approach to personalized trial design. From the relational perspective, some clinicians are fearful that the concept of trials within clinical settings will upend the nature of professionalism and the doctor–patient relationship:

It seems like it takes away the doctor’s doctoring so that the doctor becomes this scientist. You come to see your doctor because you want their opinion, and [instead] the doctor’s response is: “Well, I don’t really know. Let’s try these two things. I don’t know which one you’re going to get [first] but let’s give it a go.” So I don’t know how patients would respond to that. (Kravitz et al., 2009)

These organization- and clinician-level barriers are less relevant in nonclinical settings, where personalized trials are offered to members of the public who are seeking to manage minor symptoms or enhance well-being on their own. These trials must appeal to potential participants but can, as long as certain ethical and legal shoals are avoided, safely bypass clinicians and institutions. The prototypical example is the quantified self (QS), which is a loosely organized affiliation of self-trackers and toolmakers who who share an interest in “self-knowledge through numbers” (n.d.-l). Recent health-related projects featured on the QS website include tracking blood oxygen on Mount Everest, mindfulness following meditation, home-monitored blood glucose and lipids in response to diet and exercise, and allergic symptoms in response to different grasses. QS encourages interested parties to carry out their own projects or to join ongoing ones. They attract individuals curious about this form of self-tracking largely through word-of-mouth. For more formal projects in which the goal is to enroll a group of individuals slated to participate in similarly structured personalized trials, more robust recruitment methods are needed (Kravitz et al., 2020).

## 4. Emerging Technologies and Approaches

The main message of this review is that increased uptake of personalized trials will require strategies that maximize real and perceived benefits and minimize costs and burdens to participants. Some of these strategies are listed in Table 2.

### 4.1. Enhancing Benefits

There are six ways to enhance benefits (see Table 2). First, whether treatments are pharmacologic or non-pharmacologic, they should be as well defined as possible, have rapid onset and offset, and be convenient to administer. Tracking adherence (using the least-burdensome methods possible) is also important in helping to bifurcate the analysis into ‘intention to treat’ and ‘as treated.’ (The ‘as treated’ analysis may be of interest to some patients—and many investigators—as an indicator of expected treatment effects when adherence is at or above a preselected target.)

Second, it may be valuable to extract information as early in the trial period as possible so the most promising treatment(s) can be assigned more frequently. For example, in a trial designed to run through four pairs of crossovers (e.g., ABBAABBA), if the initial two pairs show a consistent big advantage of treatment A, then the probability of assignment to AA (rather than AB or BA) in subsequent pairs could be adjusted upward. Applying such an adaptive design may reduce the chance of being stuck on an ‘inferior’ treatment, so as to improve patient outcomes even during the trial, and thereby be more attractive for patients.

Third, it will be desirable to enhance the precision of results by extending trial length, taking more measurements (daily, hourly, or even continuously), and/or adopting measurement instruments (e.g., psychometric scales, bodily sensors) with high reliability and validity. Psychometric scales always feature a tradeoff between reliability and convenience (longer scales are, all else equal, more reliable), but computerized adaptive testing built into mobile devices holds promise for achieving greater measurement stability with fewer items (Morris et al., 2017).

Fourth, results should be reported promptly, as enthusiasm for integrating the results may decay rapidly after trial completion. Rapid turnaround can be accomplished either through shared human resources (e.g., a statistical team that is available most days of the week to perform analyses and return results with minimal delay) or automated analysis using fixed algorithms or machine learning.

Fifth, no one should assume that results will be quickly, accurately, and meaningfully interpreted by trial participants; statistical illiteracy is a widespread problem (Gigerenzer et al., 2007). Beyond that, in one qualitative study, the majority of personalized trial participants preferred simple displays (such as bar charts) that do not represent uncertainty, while a substantial minority (23%) preferred more comprehensive displays with error bands for the margin of error as the “most helpful for decision-making” (Whitney et al., 2018). Therefore, we believe it is important for personalized trials to provide flexible options for delivering the results of individual trials, allowing end users to choose their preferred format, whether it be simple or comprehensive; graphical, tabular, or textual; and with or without representation of uncertainty. A one-size-fits-all approach, providing everyone with the same comprehensive display, is unlikely to satisfy many. But providing everyone with simple bar charts might be equally dismaying to the roughly one quarter of end users who are comfortable with representations of probability and uncertainty.

Lack of flexibility (personalization) of results reporting could be one reason that the influence of personalized trial results on patients’ subsequent treatment preferences is relatively weak (Kravitz et al., 2021). Furthermore, in the context of personalized trials, the clinical significance of small differences in means has not been established for most quality-of-life measures (Jaeschke et al., 1991). Although standards for visualization and statistical analysis have been proposed (n.d.-m)(n.d.-n), much more work on identifying best practices for communicating results to users is needed (see the companion article in this issue, (n.d.-o), for further discussions, both for personalized trials and for the broader framework on personalized data science).

Finally, investigators must own up to the fact that, for many patients, personalized trials offer a *pro tem* solution. New treatments come on the market, clinical conditions evolve, comorbidities develop, and patient preferences change. New questions arise about combinations of treatments heretofore evaluated singly. Therefore, to maximize the benefits delivered, trial platforms should be flexible enough to allow for ongoing (iterative) comparisons, perhaps using information already acquired as Bayesian priors.

### 4.2. Reducing Costs and Burdens

As for reducing costs and burdens of personalized trials, we have identified five targets (see Table 2). Any trial initiated by clinicians or investigators at an academic institution or health system will require oversight by an IRB. If oversight is conducted with a light touch (particularly for trials that fall into the category of clinical care or quality improvement), implementation can proceed apace; if heavy handed, long delays and high burden can be expected. Several groups have proposed classification schemes or algorithms for identifying trials requiring greater scrutiny (n.d.-p)(Stunnenberg et al., 2020).

Another way to reduce costs and barriers is to make it easier for individuals to find relevant trials (or to design their own), complete enrollment procedures, and provide meaningful informed consent. A number of electronic platforms, many created for use on mobile devices, have been used or are in development (Barr et al., 2015)(Bobe et al., 2020)(Konigorski et al., 2020)(Kravitz et al., 2020). As (n.d.-q) point out, however,

For clinicians interested in embedding N-of-1 trials in their clinical practice, a personalized trial platform needs to be developed that allows users to customize trial designs according to the use case. A shared service that delivers custom-built trial prototypes, uses a dedicated pharmacy, and facilitates data collection and analyses might best reduce logistical and cost barriers to widespread implementation. Over time, such infrastructure can foster the development of successful supporting services and mobile health applications that both facilitate N-of-1 trials and reduce technical barriers and implementation costs.

A third target is to automate trial implementation by streamlining delivery of the intervention(s). For example, some groups have arranged to mail personalized trial participants their study drugs (Nikles et al., 2006)(n.d.-r). Others have created electronic prompts and instructional videos to deliver behavioral interventions (Kravitz et al., 2020).

A fourth approach to minimizing burdens is to make data collection easier. While more measurements are generally more psychometrically reliable than fewer, patient tolerance for frequent measures has its limits (Lee et al., 2020). Therefore, investigators have viewed with interest the prospect of sampling outcomes using mobile devices or special sensors. These approaches will undoubtedly gain credence as new technologies evolve, but they will also raise important privacy concerns.

Finally, burdens can be offset, if not eliminated, by providing potential participants with economic incentives. The investment could be worthwhile, especially if personalized trials are used as a prelude to authorization of expensive medications (Kravitz et al., 2008).

## 5. Conclusions

Personalized trials remain a promising strategy for individualizing care under conditions of increased therapeutic precision. They have focused applicability within health and medicine and, though not for everyone, they have already demonstrated broad appeal within certain populations. However, fulfilling their potential will require new approaches to maximizing benefits and minimizing burdens. Advances in biostatistics and data science, information technology, and behavioral economics hold promise for delivering personalized trials more efficiently, thereby making this ‘non-omical’ form of individualized precision medicine available to more people (n.d.-s).

## Figures and Tables

**Table 1.** Technical Requirements and Optional Elements for Personalized Trials.

**Technical Requirements Related to Population, Health Condition, and Treatment**
Heterogeneity of treatment effects
Condition that is chronic, stable, and monitorable
Rapid onset/offset of treatments
**Technical Requirements for Appropriate Design, Analysis, and Communication of Results**
Randomized or balanced assignment
Systematic outcome assessment
Framework for analysis and feedback
**Optional Elements That May Be Required in Selected Circumstances**
Blinding
Washout

**Table 2.** Strategies for Enhancing Benefits and Reducing Costs and Burdens of Personalized Trials

**Strategies for Enhancing Benefits**
Choose better comparators and maximize adherence to treatment
Use adaptive designs such as ‘play-the-winner’
Sharpen precision (with more, better, or more frequently obtained outcome measures)
Report results more quickly (using automated statistical analysis and reporting)
Report results more clearly to enhance comprehension
Make iteration (i.e., repeated trials in the same person, building on results of earlier trials) seamless
**Strategies for Reducing Costs and Burdens**
Reach consensus as to when personalized trials are ‘research’ and when they are clinical care (or quality improvement) and reduce unneeded IRB requirements
Streamline enrollment and consent procedures
Automate trial implementation (e.g., delivery of behavioral treatments or ‘nudges’)
Automate data collection (using mobile apps and sensors)
Consider economic ‘nudges’ or incentives
